# Granulomatous Lobular Mastitis Associated with* Mycobacterium abscessus* in South China: A Case Report and Review of the Literature

**DOI:** 10.1155/2017/7052908

**Published:** 2017-02-12

**Authors:** Ye-sheng Wang, Qi-wei Li, Lin Zhou, Run-feng Guan, Xiang-ming Zhou, Ji-hong Wu, Nan-yan Rao, Shuang Zhu

**Affiliations:** ^1^Guangdong Province Key Laboratory for Biotechnology Drug Candidates, School of Biosciences and Biopharmaceutics, Guangdong Pharmaceutical University, Guangzhou, Guangdong 510006, China; ^2^Sun Yat-sen Memorial Hospital of Zhongshan University, Guangzhou, Guangdong 510120, China

## Abstract

*Mycobacteria,* which are known as rapidly growing bacteria, are pathogens that are responsible for cutaneous or subcutaneous infections that especially occur after injection, trauma, or surgery. In this report, we describe a species of* Mycobacterium abscessus* that was isolated from a breast abscess in a patient who was previously diagnosed with granulomatous lobular mastitis (GLM). This current case is the first ever presented case of GLM associated with* M. abscessus* documented in South China. The case presentation highlights the role of* M. abscessus* in GLM. The association of* M. abscessus *and GLM is discussed and a summary of breast infection due to* Mycobacteria* is given.

## 1. Introduction

Granulomatous lobular mastitis (GLM), also known as idiopathic granulomatous mastitis, was first described by Kessler and Wolloch in 1972 [1]. GLM is a rare inflammatory condition of the breast with unknown etiology and variable treatment options [2–5]. Clinically, GLM may be present as a firm, red, tender lesion that suggests the presence of an abscess [6], or as a hard mass closely resembling a malignancy [7]. Although the majority GLM cases appear aseptic [8–10], case reports of documented coinfections with* Mycobacterium abscessus* have been reported in Turkey [11] and the United States [12]. To our knowledge, the present case is the first ever presented case of GLM associated with* M. abscessus* that is documented in South China.

## 2. Case Report

A 29-year-old female from South China with a history of swelling, discharge, and chronic abscesses of the right breast for more than 10 days presented to the Department of Breast Surgery in the Second Affiliated Hospital, Sun Yat-sen University, Guangzhou, China. About one month prior, the patient had noted a painful mass in the right breast and a mini-invasive operation was performed. The patient did not have a history of fever, night sweats, weight loss, and respiratory symptoms. In addition, there was no family history of breast cancer and no personal history of tuberculosis. The patient was referred to an outpatient surgery clinic, where a breast ultrasound demonstrated a duct ectasia hypoechoic lobulated mass measuring 16.7 mm × 9.8 mm in the lower quadrant of the right breast and palpable lymph nodes in the ipsilateral axilla. Blood tests showed that full blood count, renal function, liver function, thyroid function, and blood glucose were all within normal limits. The patient was treated with a variety of antibiotics, including doxycycline, clindamycin, and amoxicillin-clavulanic acid for 10 days, without success. The patient underwent drainage of the right breast and 1 ml of purulent fluid was aspirated.

**Figure 1:**
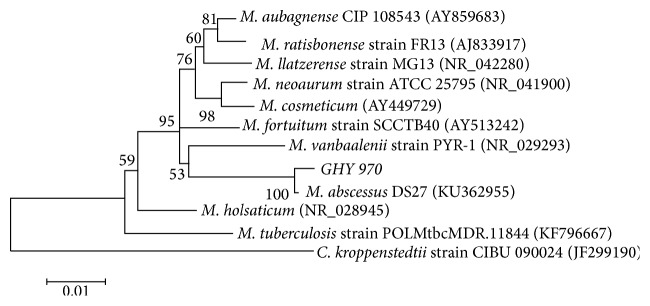
Phylogenetic tree based on 16S rDNA gene sequences of GHY 970.

Specimens were sent for routine bacteriology, acid-fast bacteria stain, Gram stain, fungal stains, and culture. Acid-fast bacilli were not found, and Gram staining of the pus showed that numerous polymorphonuclear leukocytes were present, however, without any organisms. The pus was plated on Columbia agar containing 5% sheep blood and incubated at 37°C in air supplemented with 5% CO_2_. Three days after incubation, the presence of strain GHY 970 was confirmed. The colonies that were present on the blood agar plates were identified with 16S rDNA PCR and phylogenetic analysis. Genomic DNA was extracted with the TIANamp Genomic DNA Kit (cat. No. #DP304-02), according to the manufacturer's guidelines. Bacterial 16S rDNAs were amplified by PCR using the combination of a universal primer 1492r (5′ GGT TAC CTT GTT ACG ACT T 3′) and bacterial primer 27f (5′ AGA GTT TGA TCC TGG CTC AG 3′). PCR was performed using a thermal cycler with the following cycling parameters: 95°C for 5 min as initial denaturation followed by 30 cycles of 94°C for 30 s, 55°C for 30 s, 72°C for 1 min, and 72°C for 7 min as final extension. After purification, the amplified products were sent to BGI Company for sequencing analysis. Homology search was performed using BLAST (https://www.ncbi.nlm.nih.gov) and the differences in nucleotide sequences between various bacteria were determined using the sequence alignment editor “BioEdit”. Products were further analyzed by MEGA 6.0. The Neighbor-Joining (NJ) tree was constructed using the Kimura-two-parameter (K2P) distance model. Phylogenetic analysis of the 16S rDNA gene sequence of strain GHY 970 revealed the species of* M. abscessus* (Figure 1). NCBI data search indicated >99% sequence identity to the* M. abscessus* DS27 (KU362955). Because the patient's treatment with antibiotics was discontinued, a new breast nodule appeared in the right breast, and the patient was treated with a combination of rifampicin, isoniazid, and pyrazinamide. After 3 months of treatment, the mass in the right breast and axillary lymph nodes had totally disappeared. The entire treatment therapy was completed by 6 months and, at follow-up after 1 year, there were no signs of recurrence or any other issues.

## 3. Discussion

*M. abscessus*, a rapidly growing type of* Mycobacterium*, is abundant in natural and processed water sources as well as in sewage and soil.* M. abscessus* primarily affects pulmonary, soft tissue and causes disseminated infections [13].* M. abscessus* may be present in cases following trauma or surgery after contamination of the wound, medical device implantation, and injection site abscesses.* Mycobacteria* encompass a broad range of Gram-positive bacilli that are often part of the skin microbiome. Therefore, trying to distinguish between colonization, infection, and contamination is challenging. GLM caused by* M. abscessus* is extremely rare and only occurs in few cases. We have summarized several cases of breast infection that are due to other* Mycobacteria* after nipple piercing: four cases of* M. fortuitum* [14–17] and one case of either* M. holsaticum, M. agricund*, or* M. brurnae* (Table 1) [18]. Taken together, breast abscesses can be caused by these organisms either before or after surgery.

In the case presented here,* M. abscessus* was isolated from breast abscess from a patient with GLM. Only few published reports that focus on* M. abscessus* associated with breast infections are available and, to our knowledge, this is the first case of GLM caused by* M. abscessus *in South China. Literature studies confirmed that* Mycobacterium* was not the only bacterial strain that associates with GLM. Previous studies in the USA and Canada showed that* Corynebacteria* are commonly detected in GLM [19, 20]. In addition, a recent study performed in China demonstrated that the predominance of* Corynebacteria*, especially* C. kroppenstedtii*, was observed in GLM [21]. In another study it was shown that breast tissue is not sterile but instead contains a diverse population of bacteria. The sources of these bacteria are unknown [22]. Thus, the* M*.* abscessus* found in this case may be one of the organisms associated with GLM. Therefore, to elucidate the pathological role of this organism in GLM, further research is warranted.

This case presentation was retrospective and, therefore, lacks a complete data set. However, we found that, in this patient,* M. abscessus *was associated with GLM. In the initial treatment approach of this patient, treatment with a variety of antibiotics was not successful. This could be due because the patient may be resistant to antibiotics. Therefore, antibiotic treatment was stopped and the patient underwent further clinical examination. It was suggested by clinicians and clinical microbiologists that the source of the* Mycobacteria* found in this case could be the nonpathogenic components of the normal microbiota of the skin flora. After the appearance of a new breast nodule, the patient was cured successfully with rifampicin, isoniazid, and pyrazinamide. Thus, this case presentation highlights the role of* M. abscessus* in GLM and should be targeted for optimal treatment therapy of GLM.

In addition to our study, eight cases of* Mycobacteria* were reviewed.* M. abscessus* and other* Mycobacteria* should be kept in mind when patients with chronic breast or soft tissue infections including recurrent breast abscess do not respond to standard antibiotic therapy. In conclusion, we report a patient with a breast abscess that is caused by* M. abscessus.* Drainage and a prolonged course of rifampicin, isoniazid, and pyrazinamide therapy were essential for successful treatment.

## Figures and Tables

**Table 1:** Cases of breast infection caused by *Mycobacteria *reported in the literature.

Case	Author, year	Age/ sex	Area (race)	Presentation	Surgery	Organism	Length of therapy	Outcome
1	Trupiano, 2001	17/f	American	Mass	Resection	*M. abscessus*	None	Complete response
2	Jacobs, 2002	35/f	German	Mass	Open biopsy	*M. holsaticum, M. agricund, M. brurnae*	10 days	Complete response
3	Lewis, 2004	29/f	American	Mass	Core needle and open biopsy	*M. fortuitum*	6 months	Complete response
4	Bengualid, 2008	17/f	American	Abscess	Incision and drainage	*M. fortuitum*	6 to 12 months	Relapse after 3 months
5	Yasar, 2011	38/f	Turks	Mass	Fine needle aspirations	*M. abscessus*	4 months	Complete response
6	Betal, 2011	51/f	Caucasian	Abscess	Incision and drainage	*M. fortuitum*	6 months	Complete response
7	Abbass, 2014	21/f	Canadian	Mass	Aspiration	*M. fortuitum*	6 months	Complete response
8	Present study, 2016	29/f	Chinese	Mass	Core needle and open biopsy	*M. abscessus*	1 year	Complete response
